# The Role of Peptides in Combatting HIV Infection: Applications and Insights

**DOI:** 10.3390/molecules29204951

**Published:** 2024-10-19

**Authors:** Naiera M. Helmy, Keykavous Parang

**Affiliations:** 1 Microbial Biotechnology Department, Biotechnology Research Institute, National Research Centre, Giza 3751134, Egypt; naiera.mohamed@yahoo.com; 2 Center for Targeted Drug Delivery, Department of Biomedical and Pharmaceutical Sciences, Chapman University School of Pharmacy, Harry and Diane Rinker Health Science Campus, 9401 Jeronimo Road, Irvine, CA 92618, USA

**Keywords:** AIDS, human immunodeficiency virus, entry inhibitors, peptides, vaccines

## Abstract

Peptide-based inhibitors represent a promising approach for the treatment of HIV-1, offering a range of potential advantages, including specificity, low toxicity, and the ability to target various stages of the viral lifecycle. This review outlines the current state of research on peptide-based anti-HIV therapies, highlighting key advancements and identifying future research directions. Over the past few years, there has been significant progress in developing synthetic peptide-based drugs that target various stages of the viral life cycle, including entry and replication. These approaches aim to create effective anti-HIV therapies. Additionally, peptides have proven valuable in the development of anti-HIV vaccines. In the quest for effective HIV vaccines, discovering potent antigens and designing suitable vaccine strategies are crucial for overcoming challenges such as low immunogenicity, safety concerns, and increased viral load. Innovative strategies for vaccine development through peptide research are, therefore, a key focus area for achieving effective HIV prevention. This review aims to explore the strategies for designing peptides with anti-HIV activity and to highlight their role in advancing both therapeutic and preventive measures against HIV.

## 1. Human Immunodeficiency Virus (HIV): Structure and Prevalence of Infection

Human immunodeficiency virus (HIV) infection is a major global health issue that causes Acquired Immunodeficiency Syndrome (AIDS), which ranks as the fourth leading cause of death worldwide [1]. Despite being one of the most well-studied viruses and the development of life-saving antiretroviral therapies (ART), nearly 38 million people worldwide are still infected with human immunodeficiency virus type 1 (HIV) [2].

There are two primary (genetically and etiologically) distinct variants of HIV: HIV-1 and HIV-2. These variants differ significantly in their disease progression and epidemiology, with HIV-1 being more prevalent and identified more quickly than HIV-2. Table 1 provides an overview of the prevalence of HIV infection (https://www.unaids.org accessed on 14 September 2024).

According to WHO in 2023, the global HIV/AIDS new infection rate is 0.17 per 1000 uninfected people. Of approximately 39 million people living with HIV (Table 1), about 25.6 million are in Africa. Eastern and Southern Africa have the highest new infection rates at 1.07 per 1000, while Southeast Asia has the lowest at 0.06 per 1000. Global HIV-related deaths total around 630,000, with Africa experiencing the highest mortality at about 380,000 deaths and the Eastern Mediterranean region the lowest at roughly 20,000 deaths.

The HIV genome encodes three structural proteins—Gag, Env, and Pol—and six accessory proteins: Tat (Transactivator of Transcription), Rev (Regulator of Expression of Virion Proteins), Nef (Negative Factor), Vif (Viral Infectivity Factor), Vpr (Viral Protein R), and Vpu (Viral Protein U). Gag is cleaved into four components: matrix (MA), capsid (CA), nucleocapsid (NC), and p6. HIV envelope protein (Env) produces two envelope proteins: surface (gp120) and transmembrane (gp41). Pol gives rise to three enzymes: protease, reverse transcriptase, and integrase [3,4].

HIV belongs to the Lentivirus genus within the Retroviridae family. Lentivirus infections are marked by a chronic disease progression, which includes a lengthy phase of clinical latency, continuous viral replication, and involvement of the central nervous system [5]. As with all retroviruses, HIV-1 packages its genome as a dimer composed of two homologous single-stranded (+) RNA molecules [6].

**Figure 1:**
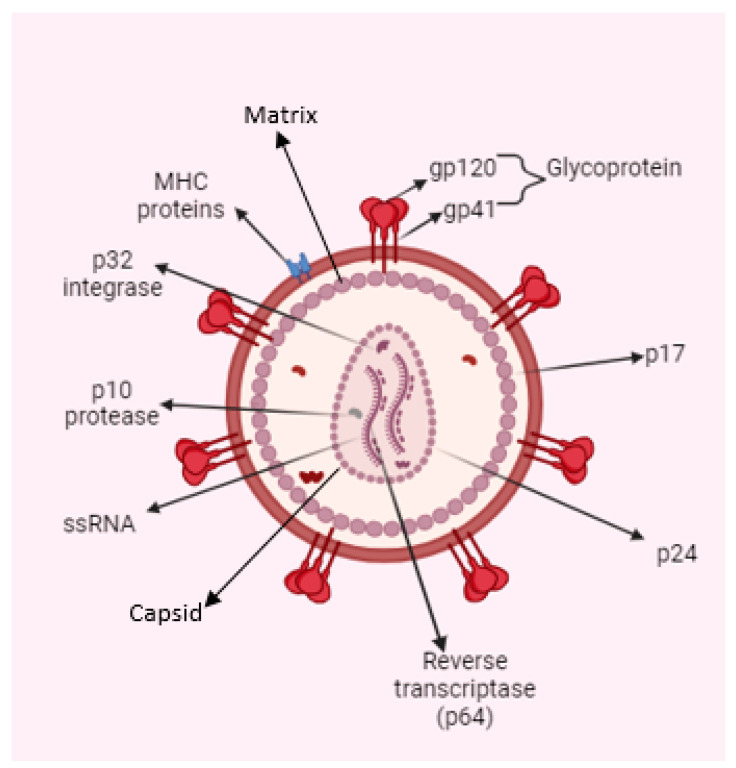
The structure and proteins of HIV. Created with BioRender.com.

The surface of HIV is marked by a unique glycoprotein called the HIV Env protein. This glycoprotein forms a trimer consisting of three heterodimer subunits not covalently linked: gp120 and gp41. The gp120 subunit is responsible for recognizing and binding to the CD4 receptor on CD4^+^ T cells and macrophages. Once bound to CD4, gp120 undergoes conformational changes that expose the co-receptor binding site, enabling interaction with co-receptors CC-chemokine receptor 5 (CCR5) and CXC-chemokine receptor 4 (CXCR4). This engagement prompts the shedding of gp120 and induces conformational alterations in gp41, which facilitates the fusion of the viral and host cell membranes, allowing the entry of the HIV capsid and genome into the host cell. As the entry process is crucial to the HIV-1 life cycle, it represents an important target for therapeutic strategies. Figure 1 illustrates the structure and proteins of HIV [7].

## 2. Anti-HIV Agents

The eradication of latent HIV-1 infection remains a key objective in antiviral research. The discovery of zidovudine, the first anti-HIV-1 drug, in 1987 marked the beginning of a new era in HIV treatment. Since then, advancements in antiviral therapies have transformed AIDS from a deadly syndrome to a manageable chronic condition. This progress is attributed to the development of various drugs that inhibit essential processes in the HIV-1 life cycle, such as reverse transcriptase, integrase, entry (attachment, fusion, post-attachment), protease, and capsid (CA) functions, as well as agents that enhance drug pharmacokinetics.

Current anti-HIV drugs target various stages of the viral life cycle. This strategy focuses on inhibiting HIV-related proteins, such as HIV-1 integrase (HIV-1 IN) (e.g., carbotegravir, dolutegravir, raltegravir), HIV-1 reverse transcriptase (HIV-1 RT) (e.g., abacavir, lamivudine, emtricitabine, tenofovir disoproxil fumarate, doravirine, efavirenz), and HIV-1 protease (HIV-1 PR) (e.g., darunavir, ritonavir, fosamprenavir, atazanavir, tripnavir). These proteins play crucial roles in the virus’s ability to infect host cells, replicate, and sustain the infection, making them key targets for therapeutic intervention [8].

The current standard of care, known as combination antiretroviral therapy (cART), ART, formerly as highly active antiretroviral therapy (HAART), involves the use of these drugs in combination to effectively manage HIV infection [9]. Some commonly used agents in HAART include drugs that target reverse transcriptase, integrase, and protease enzymes (e.g., Kaletra, Combivir, Truvada, Odefsey, Stribid, Genvoya, Symfi).

Currently, cabotegravir, an effective integrase strand transfer inhibitor, is available in two forms: as an oral tablet for daily use and as a long-acting injectable nanosuspension. The long-acting injectable form, administered intramuscularly or subcutaneously, has a half-life of approximately 40 days. This extended half-life allows for administration once a month or even less frequently [10].

There are several types of HIV inhibitors targeting different stages of the viral entry process. Fusion inhibitors, like enfuvirtide, prevent HIV from entering CD4 T lymphocytes by blocking the fusion of the virus with the host cell membrane. CCR5 antagonists, such as maraviroc, block the CCR5 co-receptors on the surface of immune cells, preventing the virus from binding and entering the cells. Fostemsavir is an attachment inhibitor that binds to gp120, obstructing HIV’s ability to enter CD4 cells. Lenacapavir, a capsid inhibitor, disrupts the HIV capsid, which protects the virus’s genetic material and essential enzymes required for replication [11].

Despite the effectiveness of cART in inhibiting the replication of HIV-1, this treatment has not achieved complete viral eradication. Low levels of latent HIV-1 provirus persist in quiescent memory CD4^+^ T cells [12]. Moreover, while cART significantly improves survival rates for HIV patients, it does not fully eliminate the virus. Persistent low-level viremia in the brain can lead to neuroinflammation through glial cells (microglia and astrocytes), potentially resulting in HIV reactivation and neuronal damage [13,14]. Thus, there remains a critical need for new strategies aimed at eradicating the latent HIV.

## 3. Peptide-Based Anti-HIV Agents

Peptides are short chains of up to 50 amino acids linked together by amide bonds, known as peptide bonds. These peptide bonds can be formed either chemically or through biological processes. Chemical synthesis allows for incorporating D-amino acids or chemically modified amino acids, which can enhance peptide stability against proteolytic cleavage. In contrast, in vitro, peptide bond formation occurs after cloning and expression of the peptide in a controlled environment. During in vivo protein biosynthesis, ribosomes integrate natural L-amino acids into nascent peptide chains [15]. This review focuses on peptide-based entry inhibitors, vaccines, RNA-targeting peptides, capsid inhibitors, defensins, and plant-derived anti-HIV peptides. However, peptide-based anti-HIV protease inhibitors, which have been developed through rational design, are not covered here as they have been extensively reviewed elsewhere [16,17].

Modulating peptide helicity and hydrophobicity can enhance their specificity as anti-HIV agents. Both amphipathicity and hydrophobicity are crucial factors affecting the antiviral activity of peptides. Peptide analogs with dimerized structures in aqueous environments, which can adopt a more helical conformation in hydrophobic conditions, have shown improved antiviral efficacy and specificity. For instance, peptides such as I6L (S-W-L-R-D-LW-D-W-I-C-E-V-L-S-D-F-K), I10L (S-W-L-R-D-I-W-D-W-L-C-E-V-L-S-D-F-K), and V13L (S-W-L-R-D-I-W-D-W-I-C-E-L-L-S-D-F-K) have demonstrated significant anti-HIV activity with IC_50_ values of >5.00 µM, 1.84 ± 0.16 µM, and 2.76 ± 0.19 µM, respectively. These peptides exhibited lower helicity in aqueous environments but higher helicity in hydrophobic conditions. The increased dimerization observed in aqueous solutions likely facilitates the formation of pores or channels during viral membrane entry, leading to membrane disruption and viral inhibition [18].

### 3.1. Peptide-Based Entry Inhibitors

The envelope surface of HIV-1 is a glycoprotein with a molecular weight of 160 kDa (gp160), crucial for binding and facilitating viral entry into host cells. The gp160 gene, after translation, is cleaved into two noncovalently associated receptor-binding subunits, gp120, and a fusion protein subunit, gp41. Transmembrane gp41 anchors Env to the membrane and associates non-covalently with gp120, thereby forming a stable trimer of heterodimers, the metastable Env prefusion conformation. When gp120 binds to the cellular receptor CD4 and a chemokine co-receptor, it initiates intricate conformational changes in gp41, ultimately resulting in the fusion of viral and cellular membranes.

**Figure 2:**
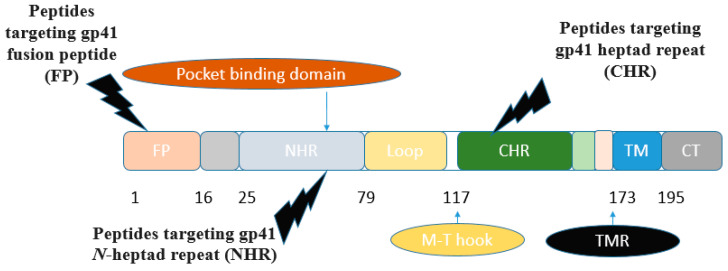
HIV-1 gp41 peptide target sites.

The ectodomain of gp41 consists of several functional regions: the *N*-terminal fusion peptide, heptad repeat 1 (HR1, *N*-heptad repeat, *N*-terminal helices (NHR)), and heptad repeat 2 (HR2, *C*-terminal helices (CHR)), along with the transmembrane region (TMR). The fusion-active (fusogenic) conformation of gp41 is a six-helix bundle, where three NHR assemble into a central trimeric coiled coil, while three CHR interlock in an antiparallel fashion within the hydrophobic grooves of this coiled core [19] (Figure 2). *N*-peptides assemble into a central trimeric coiled coil (*N* trimer), forming grooves where *C*-peptides can bind. The *N*- and *C*-terminal heptad regions of gp41 are attractive targets for designing anti-HIV agents [19,20]. Concerning peptide inhibitors, the majority of synthetic peptides investigated for targeting the entry stage of the HIV-1 life cycle originate from various domains of the HIV-1 glycoprotein gp41.

**Figure 3:**
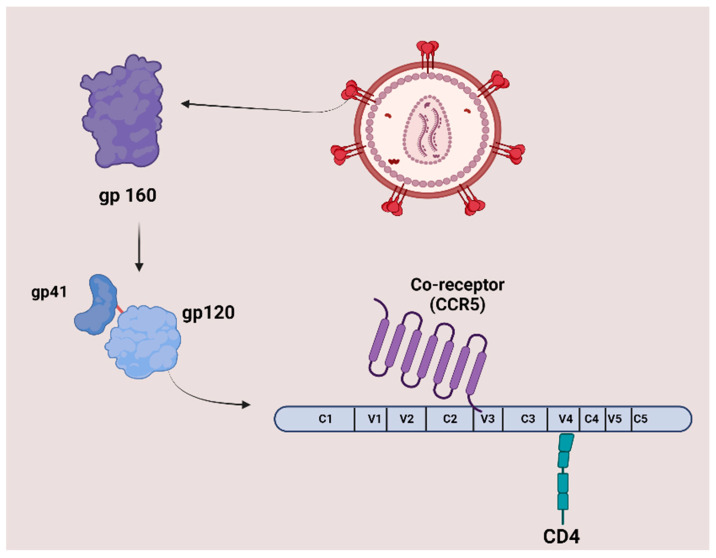
The steps of gp120 emergence for the initiation and regulation of HIV-1 infection. Glycoprotein gp160 is cleaved into gp120 and gp41 despite being attached in a trimeric form on the viral surface. The gp41 *C*-terminal subunit undergoes a conformational change necessary for viral fusion, while the gp120 *N*-terminal subunit extends outside the viral membrane. Gp120 can be structurally organized into five conserved regions (C1–C5) and five variable regions (V1–V5). The interaction between the host receptor CD4 and specific residues in the conserved regions of gp120, along with the co-receptor CCR5 binding to a GPGR/Q motif, is critical. The variable regions of gp120 and a significant amount of carbohydrates contribute to extracellular interactions and cover the protein’s surface [22]. Figure 3 was created with BioRender.com.

The gp120 subunit features five conserved constant regions (C1–C5) and five variable regions (V1–V5), which play essential roles in initiating and regulating HIV-1 infection. The interaction between the host receptor CD4 and specific residues in the conserved regions of gp120 occurs on either side of the V4 region (Figure 3).

Although CD4 was quickly recognized as the ‘primary receptor’ for the AIDS virus, it soon became evident that other molecules might also play a role. CCR5 and CXCR4 are also essential for facilitating the necessary conformational changes during the early stages of the viral cycle. Both CCR5 and CXCR4 are chemokine receptors structurally related to one another and belong to the superfamily of seven-transmembrane G-protein coupled receptors (GPCRs) [21].

The co-receptor CCR5 interacts with gp120, particularly with a GPGR/Q motif located at both the apex and base of the V3 loop. The spike-exposed surface of gp120 is primarily characterized by its variable regions and a high density of carbohydrates. These highly variable regions and the carbohydrate coating contribute to the protein’s extracellular interactions [22].

Various antibodies have been developed to target these conserved regions, including the V3 glycan attached to the N332 glycoprotein, the CD4-binding site (CD4bs), and the V1–2 loops. Key interactions include the binding of the gp120 V3 loop to the chemokine binding pocket on CCR5 and the engagement of the CD4-induced bridging sheet of gp120 with the *N*-terminus of CCR5. This structural information is crucial for designing optimized inhibitors [23].

**Figure 4:**
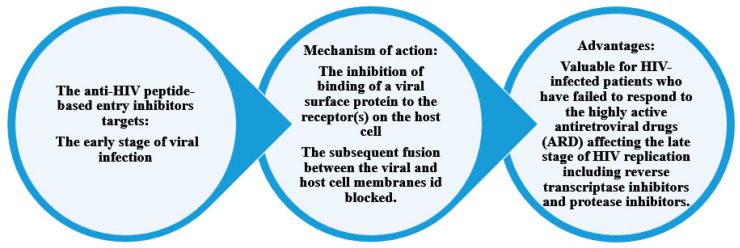
Anti-HIV peptide-based entry inhibitors.

Figure 4 shows the mechanism of action and the advantages of peptide-based HIV entry inhibitors, which interact with various sites on either viral surface proteins or cellular receptors. These include peptides that target the CD4-binding site on gp120, co-receptors, the co-receptor-binding site on gp120, the fusion peptide (FP) in gp41, and peptides that target the CHR and NHR regions in gp41. These inhibitors act in the early stage of viral infection. Additionally, other peptide-based anti-HIV strategies have been developed to neutralize cell-free virions [24].

#### 3.1.1. Peptide-Based HIV Entry Inhibitors Targeting gp120 or gp41

**Figure 5:**
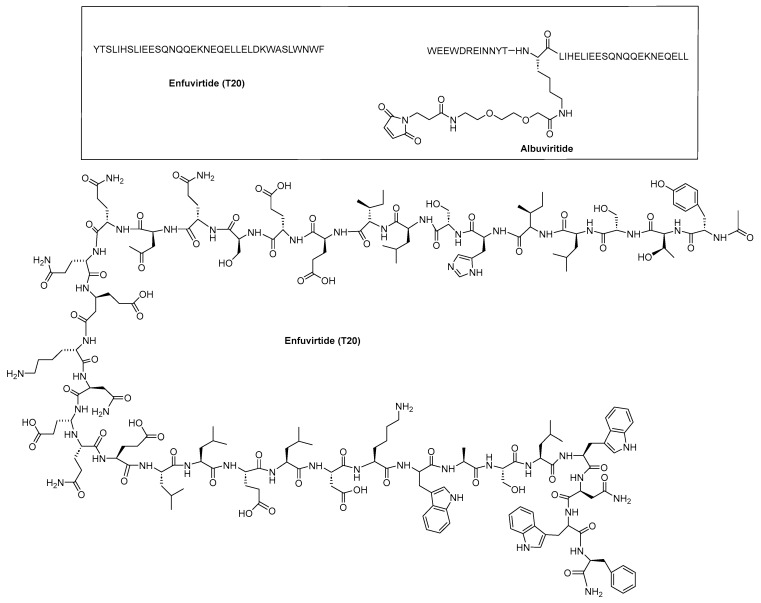
Enfuvirtide (ENF) and albuvirtide: peptide-based drugs designed to inhibit HIV entry. The chemical structure of ENF is shown in detail.

The roles of gp120 and gp41 in the HIV lifecycle have greatly influenced the development of anti-HIV peptides. The U.S. Food and Drug Administration (FDA) approved enfuvirtide (ENF, T-20, Fuzeon) and albuvirtide as peptide-based anti-HIV drugs in 2003 and 2018, respectively (Figure 5). ENF, the first peptide drug to inhibit HIV entry, was approved for patients unresponsive to other antiretrovirals [23]. It works by binding to the gp41 protein on the HIV envelope, blocking the required conformational changes for membrane fusion and viral entry [25,26]. ENF binds to helical regions in the viral gp41 protein, obstructing the formation of the six-helix bundle essential for the fusion of the viral and host cell membrane. ENF is a *C*-peptide that binds to a portion of the *N*-trimer groove, preventing the formation of the six-helix bundle in a dominant-negative manner. More specifically, ENF attaches to the first heptad repeat (HR1, *N*-helix) within the gp41 subunit of the viral envelope glycoprotein. Effective against various HIV-1 strains but not HIV-2, ENF requires high dose twice-daily intravenous administration due to its short half-life [7]. It can cause uncomfortable injection site reactions and is quite expensive [9].

The pharmacokinetics of ENF were improved through chemical glycosylation. This approach leverages the protective effects of glycosylation against protein denaturation and protease degradation. Glycosylated ENF, specifically with sialic acid (SL-ENF), demonstrated activity against HIV [27]. Additionally, to address ENF’s poor aqueous solubility, it was conjugated with polyethylene glycol (PEG). This conjugation resulted in increased solubility and an extended half-life while maintaining similar antiviral activity against HIV-1 (EC_50_ = 6–91 nM). The PEGylated form showed a high affinity for a functional domain of the HIV gp41 protein, effectively inhibiting the gp41-mediated fusion between the virus and host cell membranes [28].

Conversely, albuvirtide (ABT, FB006M) is another HIV-1 fusion inhibitor that functions as a long-acting fusion inhibitor by attaching to the transmembrane glycoprotein gp41 on the outer membrane of HIV. ABT is a peptide modified with 3-maleimidopropionic acid, and it is derived from the *N*-terminal sequence of HIV-1 gp41. It prevents replication by disrupting the fusion between the virus and the host cell membrane. ABT binds to the HR1 domain of gp1 [29] and has a significantly longer half-life compared to T20, which requires administration through injections twice daily. ABT demonstrates broad-spectrum anti-HIV-1 activity in vitro, with a half-maximal inhibitory concentration (IC_50_) ranging from 0.5 to 5.0 nM. It has proven effective against 28 distinct clinical isolates of HIV-1 in China, exhibiting IC_50_ values between 1.3 and 18.1 nM [30].

Several other peptide sequences have been investigated (Table 2) to interfere with HIV entry and effectively inhibit HIV infection. Pu et al. (2019) [23] have reviewed some of these peptides. We have organized the classes and extracted several sequences in addition to the previously reported peptides.

Various peptide-based inhibitors target sites like gp120 CD4-binding site (CD4bs), co-receptor binding site (CoRbs), and gp41 regions such as fusion peptide (FP), *N*-heptad repeat (NHR), and heptad repeat (CHR) (Figure 2). For instance, CD4M33 and M48U1 (Table 2) target the CD4bs on the HIV-1 envelope with IC_50_ values of 424.7 and 0.71 nM, respectively. The main peptide-based HIV entry inhibitors targeting gp120 or gp41 are discussed below.

##### Inhibitors Targeting gp41 *N*-Heptad Repeat (NHR) Interactions

The conserved *N*-heptad repeat (NHR) region of gp41, which is formed after binding between the CD4 receptor and HIV-1 envelop spike (Env), is one of the main targets of designed peptide inhibitors. Some of the reported gp41 NHR inhibitors peptides are SJ-2176, T20, C34, SFT, SC29EK, T1249, T1144, FB006M (ABT), CP32M, HP23, AP3, HP23-E6-IDL, YIK-C16, and MT-WQ-IDL (Table 2) with IC_50_ values of 101.0, 29.5, 12.5, 50, 9.6, 3.4, 13.9, 2.7, 65.0, 4.7, 19, 0.75, 0.09, and 2.7 nM, respectively. Also, LP11, LP40, LP46, LP52, and C34Chol (Table 2) were screened as gp41 NHR inhibitor peptides and showed IC_50_ values of 0.83, 4.29, 0.08, 0.017, and 15.5 nM, respectively.

The synthetic peptide C34 has the amino acid sequence WMEWDREINNYTSLIHSLIEESQNQQEKNEQELL (see Table 2) and features *N*-terminal acetylation and *C*-terminal amidation for enhanced stability. Two PEGylated variants of C34, PEG2kC34, and PEG5kC34, were tested for their anti-HIV properties. Their effectiveness in inhibiting HIV-1 Env-mediated cell–cell fusion was evaluated using CD4 and co-receptor-expressing TZM-bl cells as targets and HIV-1 Env-expressing HEK293T cells as effectors. The findings revealed that PEGylated C34 peptides demonstrated broad-spectrum anti-HIV-1 activity with a prolonged half-life, achieving EC_50_ values of approximately 36 nM for both PEG2kC34 and PEG5kC34. This indicates that PEGylation could be a promising strategy for developing long-acting peptide-based anti-HIV drugs [31].

He et al. (2023) [32] developed lipopeptide-19 (LP-19) with a sequence of EMTWEEWEKKVEELEKKIEELLK-PEG8-K(C16) as a broad-spectrum anti-HIV fusion inhibitor, effective against HIV-1 and HIV-2, with a high resistance barrier. This peptide was designed by adding a fatty acid group, palmitic acid (C16), to the *C*-terminus of the peptide 2P23, aiming to target a highly conserved pocket site on the HIV-1 gp41 protein using the M-T hook structure (Figure 2). The IC_50_ values of LP-19 against various HIV subtypes were 0.76 nM for subtype B, 0.29 nM for CRF01_AE, 0.38 nM for CRF07_BC, 0.85 nM for CRF08_BC, and 0.44 nM for URF [32].

**Figure 6:**
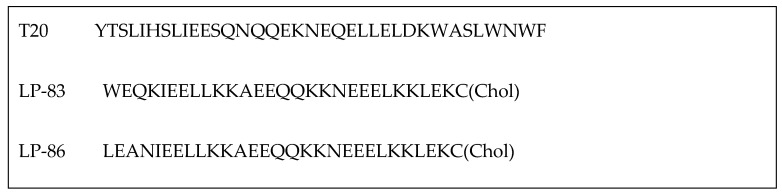
Sequences of LP-83 and LP-86.

As described earlier, ENF (Figure 5) is a peptide drug used in combination therapy to treat HIV-1 infection, acting as a membrane fusion inhibitor. While ENF has proven effective in combination therapies, its limited antiviral activity and the potential for resistance have driven efforts to enhance its efficacy. Recent research has focused on conjugating T-20 backbone-based fusion inhibitors with lipids to boost their anti-HIV activity. For example, cholesterol-conjugated peptides LP-83 and LP-86, depicted in Figure 6, demonstrated strong inhibitory effects against various HIV-1 strains, including HIV-1NL4-3 (X4 tropic), HIV-1JRCSF (R5 tropic), and HIV-189.6 (R5X4 tropic), with IC_50_ values of 0.49 ± 0.03, 4.54 ± 1.42, and 4.75 ± 0.83 pM for LP-83, and 0.43 ± 0.03, 4.78 ± 0.69, and 7.24 ± 1.36 pM for LP-86, respectively [33].

Short peptide-based inhibitors of HIV-1 fusion have been developed by creating a series of m-xylene thioether-stapled 22-residue α-helical peptides using a dithiol bisalkylation reaction. These peptides target the HIV-1 glycoprotein gp41 and act as fusion inhibitors. By focusing on the helix-zone binding domain of the gp41, researchers successfully synthesized stapled peptides, including one named hCS6ERE. The peptide Hcs6ERE has the sequence IEELI^A AQ^QQRK NEEALRE L, where ^ indicates the positions of homocysteine residues that form staples upon reaction. This peptide demonstrated potent inhibition of HIV-1, with an EC_50_ of 7.2 ± 0.3 nM, effectively blocking Env-mediated cell–cell fusion and viral replication. Furthermore, combining Hcs6ERE with another fusion inhibitor, HP23, which targets the NHR region, resulted in a synergistic anti-HIV-1 effect. Both peptides target the gp41 NHR, with HP23 overlapping a six-amino acid sequence at the *N*-terminus of Hcs6ERE. HP23 features an M-T hook structure and a PBD sequence, primarily binding to the pocket region on the gp41 NHR [34].

In a separate study by Mzoughi et al. (2019) [35], two trimeric synthetic peptides—N36 (546SGIVQQQNNLLRAIEAQQHLLQLTVWGIKQLQARIL581) and C34 (628WMEWDREINNYSTLIHSLIEFSQNQQEKNEQELL661)—were evaluated. These mimicked the *N-* and *C*-terminal heptad repeat HR1 and HR2 domains, which are essential for HIV-1 entry through membrane fusion. The research showed that trimerization of the N36 peptide enhanced both its stability and antiviral activity, whereas trimerization of the C34 peptide did not significantly improve its stability or efficacy. The improved performance of the trimeric N36 peptide was attributed to its *C*-terminal region better mimicking the flexible orientation of this region in the HIV-1 gp41 structure. Conversely, the addition of two amino acids, arginine and glutamic acid, at the *C*-terminal end of the C34 peptide (C34 RE) enhanced its activity. Additionally, the study found that coupling trimeric peptides with various carriers capable of inducing coiled-coil trimerization and maintaining α-helix structures preserved, or even enhanced, their antiviral activity compared to monomers. These results underscore the potential of trimeric peptides as HIV-1 inhibitors and suggest promising directions for developing peptide-based fusion inhibitors [35].

In summary, peptide inhibitors targeting the NHR region of HIV-1 gp41 have demonstrated significant potential in blocking viral entry and fusion. Recent advancements have significantly enhanced the efficacy and stability of these inhibitors. For instance, PEGylated versions of the C34 peptide, such as PEG2kC34 and PEG5kC34, showed broad-spectrum anti-HIV-1 activity and prolonged half-lives, highlighting PEGylation as a promising strategy for developing long-acting anti-HIV drugs. Additionally, lipopeptide-19 (LP-19) and cholesterol-conjugated peptides LP-83 and LP-86 exhibit inhibitory effects against multiple HIV-1 strains, suggesting that lipid conjugation can further enhance peptide efficacy. Overall, these developments underscore the continuing relevance of peptide inhibitors targeting the NHR region of gp41. Innovative approaches, such as the use of stapled peptides and trimeric constructs, have also shown promising results. Stapled peptides like Hcs6ERE exhibit strong antiviral activity, while trimeric peptides, such as N36, demonstrate improved stability and efficacy due to their structural mimicry of the HIV-1 gp41 heptad repeat regions. The combination of enhanced peptide stability, novel conjugation techniques, and strategic structural modifications paves the way for more effective and durable anti-HIV-1 therapies.

##### Inhibitors Targeting gp41 Heptad Repeat (CHR) Interactions

A number of peptides were also based on the carboxy-terminal leucine/isoleucine heptad repeat (CHR) region of gp41 (Table 2). Peptides T21/DP107 [36,37] and IZN17 [38] (Table 2) inhibited the gp41 CHR region with IC_50_ values of 320 and 22 nM, respectively.

Several other peptide inhibitors targeting HIV-1 entry were designed to match the *C*-terminal heptad repeat of the HIV-1 gp41 protein (*C*-peptides). These peptides function by binding to the central coiled-coil region of gp41 in a helical conformation, acting in a dominant-negative manner. Among these, C14linkmid and C14Aib [39] (Table 2), which showed minimal helical content or propensity, exhibited inhibitory potency with IC_50_ values of 35 µM and 144 µM, respectively, and strong binding affinity to the gp41 hydrophobic pocket. The effectiveness of these peptides is significantly influenced by the position of the crosslinker within the peptide sequence. An *N*-terminal crosslinker, for instance, incurs a greater enthalpic penalty, leading to reduced binding affinity. The incorrect positioning of crosslinkers, such as the misalignment of Trp-628 and Trp-631, which are key pocket-binding residues, also impacts efficacy. Specifically, Trp-628, being outside the linker of C14linkmid, contributes to entropic stabilization by hindering the crosslinker’s ability to cap the helix. Consequently, stabilization at the *N*-terminal, which is generally more flexible than the central part of the helix, results in a more significant reduction in degrees of freedom compared to stabilization in the middle of the helix [39].

Wild et al. (1994) [40] synthesized peptides based on the CHR HIV-1 gp41 protein. Peptide DP-178 was derived from residues 643–678 of the HIV-1_LA_I isolate and designed to adopt an α-helical secondary structure. The peptide inhibited the HIV life cycle at an early stage before reverse transcription. DP-178 (YTSLIHSLIEESQNQQEKNEQELLELDKWASLWNWF) demonstrated inhibitory effects against primary HIV-1 strains 596, 598, and LA1 in PBMCs, with ID_50_ (dose of peptide causing a 50% reduction in reverse transcriptase cpm) values of 2.8 µg/mL, 1.2 µg/mL, and 1.1 µg/mL, respectively. These results suggested that DP-178 exerts its antiviral effect by interfering with viral entry or uncoating, affecting early steps before provirus formation, rather than later stages involving envelope synthesis, processing, or assembly [40].

**Figure 7:**
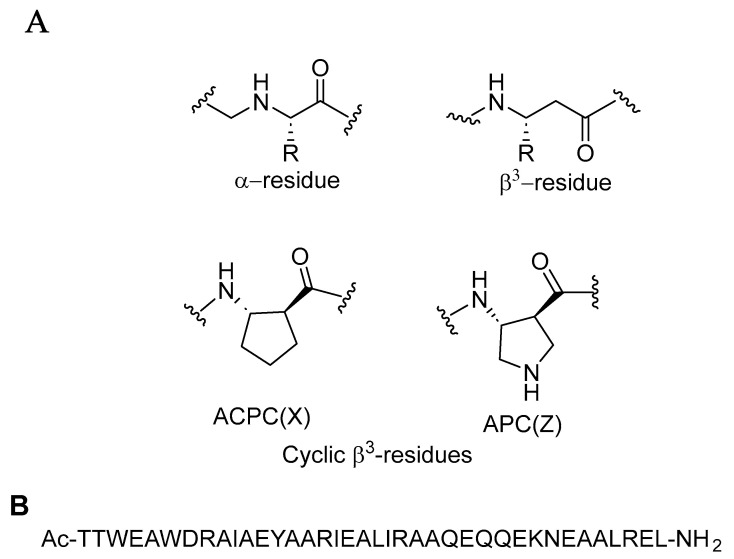
(**A**) Structures of an α-amino acid residue, the corresponding β^3^ residue analog, and cyclic β-residues ACPC and APC. (**B**) Primary sequences of α-peptides (adopted from [42]).

The combination of natural α-amino acids with unnatural β-amino acids results in a distinctive class of molecules known as α/β-peptides. These α/β-peptides possess a mixed backbone that supports a variety of folding patterns and biological functions, similar to natural peptides and proteins. Despite their unconventional structures, α/β-peptides offer notable advantages over natural peptides, such as enhanced stability against protease-mediated degradation [41]. By integrating both α- and β-amino acids into polypeptide sequences derived from the HIV protein gp41, Horne et al. (2009) [42] created molecules that effectively replicate the structural and functional properties of critical gp41 subunits. These α/β-peptides were demonstrated to inhibit HIV-cell fusion through mechanisms akin to those of gp41-derived α-peptides, highlighting their potential as inhibitors in HIV research. The α-peptide structure illustrated in Figure 7 binds tightly to gp41-5, demonstrating significant inhibition of HIV-1 infectivity, with IC_50_ values ranging from 5 ± 0.6 to 56 ± 6 nM depending on the strain. The cell–cell fusion assay results demonstrated that the two α/β-peptides, Ac-TTWEAWDRAIAEYAARIEALIRAAQEQQEKNEAALREL-NH_2_ and Ac-TTWEXWDZAIAEYAXRIEXLIZAAQEQQEKNEXALZEL-NH_2_, exhibited IC_50_ values of 7 ± 2 nM and 5 ± 2 nM, respectively. These findings indicate that these peptides effectively mimic the structural and functional aspects of gp41 through their CHR-derived α/β-peptide sequences [42].

In summary, peptides targeting the CHR region of the HIV-1 gp41 protein have shown varying degrees of effectiveness in inhibiting viral entry. While peptides targeting the CHR region of gp41 offer promising therapeutic potential, their effectiveness is closely linked to their structural design and binding characteristics. The introduction of α/β-peptides, which combine natural α-amino acids with unnatural β-amino acids, presents a promising alternative due to their enhanced stability and effective mimicry of gp41 structures. Continued optimization of these peptides, including precise crosslinker placement and structural modifications, is essential for enhancing their inhibitory potency and advancing their application in HIV treatment.

**Table 2:** Peptide-based HIV entry inhibitors targeting gp120 or gp41 interactions.

**Inhibitors Targeting gp120 CD4 Binding Site Interactions**		
CD4M33	TpaNLHFCQLRCKSLGLLGKCAGSBipCACV-NH_2_ Tpa = Thiopropionic acid; Bip = biphenylalanine.	[43]
M48U1 M48U2 M48U3	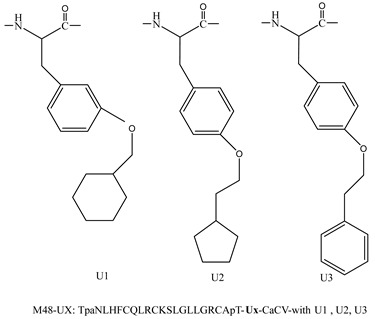	[44,45]
**Inhibitors Targeting gp41 *N*-heptad Repeat (NHR) Interactions**		
SJ-2176	Amino acid residues 637–666: EWDREINNYTSLIHSLIEESQNQQEKNEQEGGC	[46,47]
T20 (ENF)	YTSLIHSLIEESQNQQEKNEQELLEDKWASLWNWF	[48]
C34	WMEWDREINNYTSLIHSLIEESQNQQEKNEQELL	[31,49]
PEG2kC34 and PEG5kC34	PEGylated variants of C34	[31]
C34-cholesterol (C34-Chol)	C34-GlySerGly-Cys(Chol)	[50]
CP34 RE	CP34-Arg-Glu	[35]
SFT	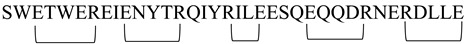	[51]
SC29EK	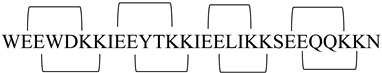	[52]
T1249	WQEWEQKITALLEQAQIQQEKNEYELQKLDKWASL WEWF	[53]
T1144	TTWEAWDRAIAEYAARIEALLRALQEQQEKNEAALREL	[54]
FB006M (ABT)	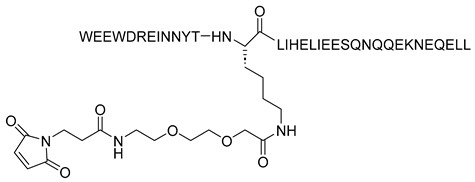	[29,55]
CP32M	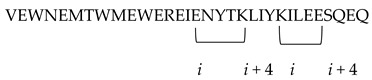 *i* to *i* + 4 position of the helical conformation.	[56]
HP23	EMTWEEWEKKIEEYTKKIEEILK	[57]
AP3	KKISEEQKKIQEEIKKILEESKKILEEIKKDWEEWIM	[58]
HP23-E6-IDL (626–656)	EMTWEEWEKKIEEYTKKIEEILKKSQNQQIDL IDL = Ile-Asp-Leu)	[59]
YIK-C16	EMTWEEWEKIEEYIKKIEEILKKSQNQQIDLGSG-PEG4-K(Palm)	[60]
MT-WQ-IDL (626–656)	MTWEEWDKKIEEYTKKIEELIKKSQNQQIDL	[61]
LP-11	EMTWEEWEKKIEEYTKKIEEILK-PEG8-K(C16)	[62]
LP-19	EMTWEEWEKKVEELEKKIEELLK-PEG8-K(C16)	[32]
LP40	YTSLIHSLIEESQNQQEKNEQELLELDK(C16)	[62]
LP46	WQEWEQKI-------TALLEQAQIQQEKNEYELQKLDK(C16)	[63]
LP52	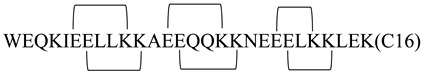	[63]
LP-83	WEQKIEELLKKAEEQQKKNEEELKKLEKC(Chol)	[33]
LP-86	LEANIEELLKKAEEQQKKNEEELKKLEKC(Chol)	[33]
Hcs6ERE	IEELI^A AQ^QQRK NEEALRE L	[34]
N36	SGIVQQQNNLLRAIEAQQHLLQLTVWGIKQLQARIL	[35]
**Inhibitors Targeting gp41 Heptad Repeat (CHR) Interactions**		
T21/DP107	Ac-NNLLRAIEAQQHLLQLTVWGIKQLQARILAVERYLKDQ-NH_2_	[36,37]
IZN17	Ac-IKKEIEAIKKEQEAIKKKIEAIEK...............LLQLTVWGIKQLQARL-NH_2_	[38]
C14linkmid	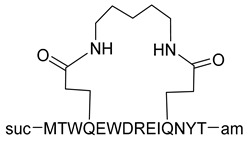	[39]
C14Aib	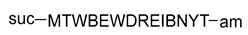	[39]
DP-178	YTSLIHSLIEESQNQQEKNEQELLELDKWASLWNWF	[40]
CHR-derived α/β-peptide sequences	AcTTWEAWDRAIAEYAARIEALIRAAQEQQEKNEAALREL-NH_2_ AcTTWEXWDZAIAEYAXRIEXLIZAAQEQQEKNEXALZEL-NH_2_	[42]
**Inhibitors Containing a Pocket-Binding Domain**		
P35A4	QEESIKKWEEWSKKIEELIKKSEELIKKIEEQIKK	[64]
PP24C	QEESIKKWEEWSKKIEELIKKIEEQIKK-PEG_24_(NH_2_-(PEG)_24_-CH_2_CH_2_COOH)-C(Chol)	[64]
PIE-12	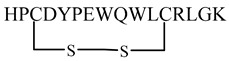	[65]
**Inhibitors Targeting Coreceptor CXCR4 or CCR5 Binding Interactions**		
pV2α-Tys	Lys-Val-Gln-Lys-Glu-Tyr(SO_3_H)-Ala-Leu-Phe-Tyr(SO_3_H)-Glu-Leu-Asp-Ile-Val-Pro-Ile-Asp	[66]
pCCR5-Tys	Met-Asp-Tyr-Gln-Val-Ser-Ser-Pro-Ile-Tyr(SO_3_H)-Asp-Ile-Asn-Tyr(SO_3_H)-Tyr-Thr-Ser-Glu-Pro-Ser-Gln-Lys	[66]
Cyclic disulfide peptides (L and D)	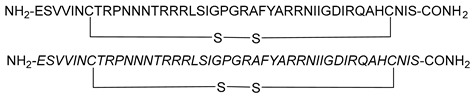	[67]
EPI-X4 JM#173-C	d-I-LRWSRKC	[68]
Trifunctional construct	SAv-VIR-102C9-EPI-X4	[68]
**Irreversible Env Inactivators**		
Macrocyclic peptide triazole AAR029b	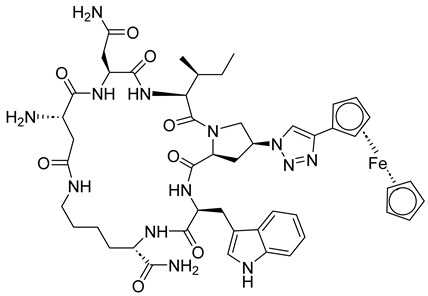	[69]
FITC-AAR029b	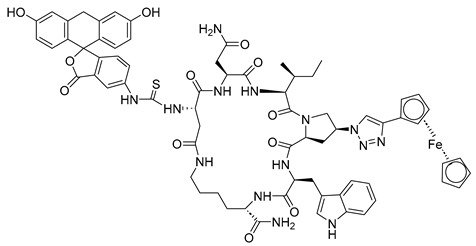	[69]
UM15	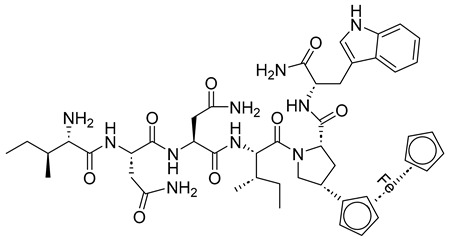	[69]
**Inhibitors Targeting gp41 Fusion Peptide (FP) Interactions**		
VIR-576 VIR-353	(LEAIPCSIPPEFLFGKPFVF) × 2 LEAIPCSIPPCFLFNKPFVF	[70,71]
E1P47 E1P47-1 E1P47-2	WILEYLWKVPFDFWRGVI ILEYLWKVPFDFWRGVIS LEYLWKVPFDFWRGVISL	[72]
**Transmembrane Protein Sequence-Derived Anti-HIV Peptides**		
P3	WQEWEQQVRYFLEANISQRLEQAQIQQEKNMYELQKLNSWDVFGNWF	[73]

##### Inhibitors Containing a Pocket-Binding Domain

The development of HIV-1 entry inhibitors has also focused on peptides containing a pocket-binding domain (PBD) designed to target the deep pocket region and adjacent subpocket sites on the *N*-trimer of the HIV-1 gp41 protein, which are critical for HIV-1 fusion. Notable examples include the artificial α-helical peptides P35A4 (QEESIKKWEEWSKKIEELIKKSEELIKKIEEQIKK) and PP24C (QEESIKKWEEWSKKIEELIKKIEEQIKK-PEG_24_(NH_2_-(PEG)_24_-CH_2_CH_2_COOH)-C(Chol)), with PP24C being cholesterol-tagged [64]. The α-helical complexes formed between PP24C and the NHR peptide T21 or N36 are comparable to those formed with the parent peptide P35A4. Specifically, the interaction between PP24C and T21 forms a heterogeneous 6HB complex. These peptides inhibit HIV-1 infection by binding to the exposed viral gp41 NHR region and disrupting the formation of the 6-HB complex between gp41 NHR and CHR. The binding of T21 to P35A4 or PP24C targets both the gp41 deep cavity and subpocket sites, effectively inhibiting HIV-1 fusion [64].

The HIV gp41 *N*-trimer pocket region is an optimal target for viral intervention due to its extracellular location, high conservation, and crucial role in viral entry. The D-peptide PIE-12 trimer binds explicitly to this pocket region of gp41. Additionally, D-peptides are more resistant to protease degradation. Welch et al. (2010) [65] discovered a pocket-specific D-peptide using phase display as PIE12 (HP**C**DYPEWQWL**C**RLGK), which contains a short core sequence surrounded by a disulfide bond between two cysteine residues that imparts structural rigidity required for binding. The analog showed IC_50_ values of 33 nM and 1100 nM against HXB2 and JRFL strains, respectively. The PIE12 monomer was generally much less potent than the PIE12 trimer. The PIE12 trimer could endure resistance mutations that affected earlier D-peptides, requiring a significantly longer selection period (65 weeks) to develop resistant strains [65].

##### Inhibitors Targeting Coreceptor CXCR4 or CCR5 Binding Interactions

HIV-1 variants rely on either CCR5 or CXCR4 for infection, with CCR5 being crucial for initial transmission and chronic infection. Maraviroc (Selzentry) is a non-peptidic approved medication that inhibits the CCR5 co-receptor on the host cell surface, but it is ineffective against HIV-1 strains that utilize the CXCR4 receptor for viral entry [74].

Inhibitors pV2α-Tys and pCCR5-Tys were identified by Cimbro et al. (2016) [66] as interacting with gp120 co-receptor binding sites (CoRbs), exhibiting IC_50_ values ˂ 50,000 and ˃50,000 nM, respectively. The tyrosine-sulfated peptide pV2α-Tys, derived from the V2 region, mimics the CCR5 N-terminus both structurally and functionally, effectively blocking HIV-1 infection. This peptide adopts a CCR5-like helical conformation, allowing it to interact directly with gp120 in a CD4-dependent manner, and competes with the CCR5 *N*-terminal peptide [66].

While sulfated V2 mimics show promise, neither sulfated nor non-sulfated V2 peptides significantly inhibited HIV-1 entry and fusion by blocking co-receptor utilization. The key factor appears to be the highly conserved *C*-terminal sulfotyrosine (Tys177), which plays a dominant role in their effectiveness. The ability of CCR5 *N*-terminal peptides and V2 mimics to inhibit a broad range of HIV-1 strains, regardless of co-receptor tropism, underscores the importance of the overall structural conservation of the gp120 co-receptor-binding site [66].

**Figure 8:**
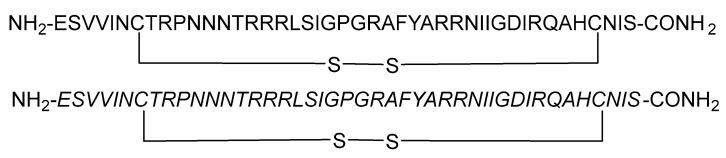
The amino acid sequence of the two L- and D- (shown in *italics*) peptides corresponds to the full length of the V3 loop of gp120 of HIV-1 89.6 strain. The cyclization is done through a disulfide bond between the two cysteines.

During HIV entry, the third variable loop (V3 loop) of the HIV-1 envelope glycoprotein gp120 binds to the CXC chemokine receptor 4 (CXCR4) co-receptor. To investigate this interaction, synthetic peptides covering the entire V3 loop were used by Zhu et al. [67]. By creating a cyclic peptide with enhanced conformational stability, the ends of the V3 loop were covalently linked via a disulfide bond (Figure 8). To further study the impact of peptide side-chain conformations on CXCR4 recognition, an all-D-amino acid analog of the L-V3 loop peptide was synthesized. Both cyclic L- and D-V3 loop peptides exhibited similar binding to the CXCR4 receptor but not to the CCR5 chemokine receptor, demonstrating selective interaction with CXCR4. These results suggest that the HIV-1 gp120 V3 loop–CXCR4 interface can accommodate ligands with different chiralities, potentially allowing the virus to maintain co-receptor recognition even with V3 loop mutations [67].

The gp41 fusion peptide has been targeted by a virus-inhibitory peptide (VIRIP), a 20-amino acid fragment derived from α1-antitrypsin. The endogenous peptide inhibitor of CXCR4 (EPI-X4), a 16-residue fragment of human serum albumin, blocks HIV-1 entry by binding to the CXCR4 co-receptor. Two promising peptides, VIRIP102C9 (sequence LEIAIPMSICEVPPNKPFVF), a fusion peptide inhibitor, and EPI-X4 JM#173 (sequence d-I-LRWSRKC), an optimized variant of EPI-X4 as CXCR4 antagonist, were evaluated for their potential. Combining these peptides into a single construct (streptavidin (Sav) [13]-VIR-102C9-EPI-X4 JM#173-C) demonstrated enhanced activity against both CCR5- and CXCR4-tropic HIV-1 variants. The study highlighted the benefits of integrating three different thiol-reactive moieties into a single system using a bis-sulfone moiety coupled with a maleimide functionality. Additionally, combining the two peptide sequences through internal amino acid modifications and conjugating them with natural amino acid side chains, along with incorporating an affinity group (biotin), facilitated the assembly of tetravalent bispecifics on a protein nanoplatform [68].

In brief, peptides containing pocket-binding domains have been designed to target the deep pocket and adjacent subpocket sites on the HIV-1 gp41 protein, which are essential for the fusion process. This approach highlights the importance of targeting both the deep cavity and subpocket sites on gp41 to inhibit HIV-1 infection effectively. Coreceptor binding site inhibitors, such as the tyrosine-sulfated peptides pV2α-Tys and pCCR5-Tys, have been identified as effective in mimicking the CCR5 *N*-terminus, thus blocking HIV-1 infection. Despite their potential, these sulfated V2 mimics have not significantly inhibited HIV-1 entry and fusion, which suggests that additional factors, such as the highly conserved *C*-terminal sulfotyrosine (Tys177), play a critical role in their effectiveness. The broader implication of these findings is the importance of the structural conservation of the gp120 co-receptor-binding site in developing effective inhibitors.

Research into the V3 loop of gp120 has led to the development of cyclic peptides with enhanced stability. Studies of these peptides have shown that they bind selectively to CXCR4, and this binding is maintained even with different chiralities of the peptides. This suggests that the V3 loop–CXCR4 interface can accommodate various ligand conformations, which might help the virus evade inhibition by maintaining co-receptor recognition despite mutations.

The development of innovative peptide constructs, such as the combination of VIRIP102C9 and EPI-X4 JM#173, has shown promise. VIRIP102C9 targets the gp41 fusion peptide, while EPI-X4 JM#173, an optimized CXCR4 antagonist, blocks the CXCR4 co-receptor. Integrating these peptides into a single construct has demonstrated enhanced activity against both CCR5- and CXCR4-tropic HIV-1 variants. This approach, which combines different thiol-reactive moieties and uses a protein nanoplatform, highlights the potential for creating multifunctional peptide-based therapies.

##### Irreversible Env Inactivators

**Figure 9:**
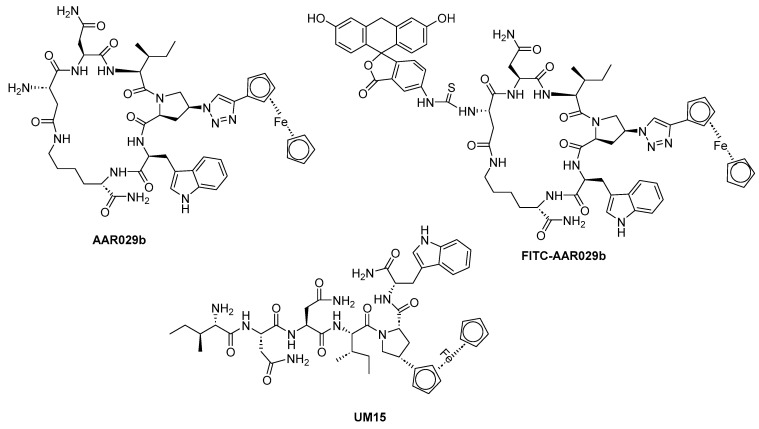
Chemical structure of cPTs AAR029b and FITC-AAR0.29b and linear hexapeptide triazole UM15.

Peptide triazoles (PTs) are a class of HIV-1 entry inhibitors that irreversibly inactivate Env trimers by exploiting the protein’s inherent metastable nature. A related group, peptide triazole thiols (PTTs), can simultaneously bind to both the CD4 receptor and co-receptor sites on the Env gp120 subunit. This dual binding induces significant conformational changes in Env, enhancing their effectiveness as inhibitors [13]. Macrocyclic peptide triazoles (cPTs) are capable of binding to HIV-1 Env gp120 with nanomolar potency, leading to irreversible inactivation of the virus. The cyclic peptide triazoles AAR029b and FITC-AAR029b, as shown in Figure 9, demonstrated antiviral activity against HIV-1 with EC_50_ values of 210 ± 10 nM and 320 ± 20 nM, respectively. FITC-AAR029b and AAR029b encapsulated in liposome formulations remained intact after 24 h of exposure to a human serum protease mixture, while the corresponding linear peptide UM15 was rapidly degraded within minutes. These findings indicate that both AAR029b and its FITC-conjugated form maintained metabolic stability under physiological assay conditions [69]. Overall, PTs exploited the metastable nature of Env trimers, and PTTs, which bind to both CD4 receptor and co-receptor sites on the gp120 subunit, enhance inhibition through significant conformational changes in Env.

##### Inhibitors Targeting gp41 Fusion Peptide (FP) Interactions

Fusion peptides (FPs) are crucial for the infection process of many viral pathogens, as they are inserted into the cellular membrane to facilitate entry. Initially, it was believed that FPs were difficult to target for therapy due to their limited accessibility. However, an optimized derivative of an endogenous α1-antitrypsin fragment, known as virus inhibitory peptide (VIRIP) (VIR-576), has shown promise by targeting the gp41 FP and effectively reducing viral loads in individuals infected with HIV-1. Münch et al. (2007) [70] indicated that FP inhibitors VIR-353 and VIR-576 significantly inhibited HIV-1 spread in ex vivo infected HLAC cultures, showing a notable reduction in viral activity at 100 nM and complete suppression of viral replication at 1 μM [70]. VIR-576 demonstrated effectiveness in a phase I/II clinical trial with a high genetic barrier to resistance. Despite this, partially resistant CXCR4-tropic HIV-1 variants emerged after extended passaging in the presence of another derivative, VIR-353, with seven mutations identified across the envelope glycoprotein but not within the gp41 fusion peptide. This indicates that the genetic barrier to HIV-1 resistance against VIRIP-based inhibitors is high [71].

A limited number of peptides have been established as fusion inhibitors that are not derived from gp41 domains. GB virus C (GBV-C) is a lymphotropic virus that infects humans and replicates in primary T and B lymphocytes. The 18-mer synthetic peptides EIP47 (WILEYLWKVPFDFWRGVI), E1P47-1 (ILEYLWKVPFDFWRGVIS), and E1P47-2 (LEYLWKVPFDFWRGVISL), derived from the GBV-C E1 protein (E1(139–156)), demonstrated antiviral activity against HIV-1 with IC_50_ values of 2.7, 5.4, and 6.6 µM, respectively, and targeted the gp41 FP [72]. The E1P47 peptide interacts with the highly conserved *N*-terminal region of HIV-1 gp41 (the FP), crucial for viral entry, in a manner similar to the inhibitor VIR576.

Overall, the findings emphasize the potential of peptide-based inhibitors, targeting the gp41 fusion peptide and incorporating innovative structural modifications, in advancing HIV-1 therapy.

##### Transmembrane Protein Sequence-Derived Anti-HIV Peptides

The reconstruction of transmembrane protein sequences from HIV-2 and simian immunodeficiency virus (SIV) led to the development of ancestral peptides derived from helical region 2 (HR2). Among these, the peptide P3 (WQEWEQQVRYFLEANISQRLEQAQIQQEKNMYELQKLNSWDVFGNWF) exhibited notable anti-HIV-1 activity, including against T-20-resistant variants, as well as against HIV-2, with low antigenicity and high stability. P3’s ability to form a typical α-helical structure in solution and its strong binding to the transmembrane protein resulted in potent inhibition of both HIV-2 (mean IC_50_ of 63.8 nM) and HIV-1 (mean IC_50_ of 11 nM), including T-20-resistant isolates. The presence of the N43K mutation in the HR1 region of HIV-1 increased P3 resistance, indicating that P3 targets the HR1 region in the transmembrane glycoprotein [73].

### 3.2. Peptide-Based Vaccines

The development of an effective HIV-1 vaccine faces multiple challenges, including the emergence of highly resistant viral strains to current anti-HIV drugs, the persistence of latent viruses, concerns about drug toxicity and interactions, and difficulties in determining the optimal administration route [75].

HIV-1’s ability to evade immune surveillance limits neutralizing humoral responses to a few vulnerable sites on the envelope glycoprotein. A promising strategy in vaccine development involves using epitope-driven vaccines targeting HIV-1.

Analysis of the primary sequence of the HIV-1 isolate CDC-451 to investigate its potential immunopathological interactions with the human proteome revealed that they share 50 heptapeptides and three octapeptides. Notably, 34 of these 50 heptapeptides have been experimentally confirmed as targets of immune responses following HIV-1 infection. This suggests that focusing on epitopic peptides with minimal similarity to the human proteome could provide a basis for developing rational anti-HIV vaccines with reduced adverse effects [76].

Among these, the membrane-proximal external region [77] of the gp41 transmembrane subunit has been identified as a critical linear B-cell epitope and a significant vulnerability site for HIV. As a result, peptide-based immunogens targeting the membrane-proximal external region [77] on gp41 have become a central focus in HIV vaccine development. These peptide vaccines consist of one or more peptide sequences (each over 15 amino acids long) that can stimulate B- and T-cells, either on their own or when conjugated with carrier proteins, scaffolds, or supramolecular complexes like liposomes [78].

Addressing the virus’s high variability, Kardani et al. [79] developed two distinct DNA constructs, pEGFP-nef-vif-gp160-p24 and pEGFP-nef-vpu-gp160-p24, and two peptide constructs, Nef-Vif-Gp160-P24 and Nef-Vpu-Gp160-P24, and complexed them with four different cell-penetrating peptides (CPPs) to facilitate delivery into mammalian cells. The CPPs included MPG (ALFLGFLGAAGSTMGAWSQPKKKRKV) and HR9 (CHHHHHRRRRRRRRRHHHHHC) for DNA delivery, and CyLoP-1 (CRWRWKCCKK) and LDP-NLS (KWRRKLKKLRPKKKRKV) for delivery of both DNA and peptide constructs. These constructs were designed to form stable, non-covalent nanoparticles at specific ratios, allowing efficient transport into cells via the CPPs. The study found that the MPG and HR9 peptides were effective at delivering DNA constructs encoding epitopes, pEGFP-nef-vif-gp160-p24 and pEGFP-nef-vpu-gp160-p24. Conversely, CyLoP-1 and LDP-NLS successfully transferred the polyepitopic peptides rNef-Vif-Gp160-P24 and rNef-Vpu-Gp160-P24 into cells. Despite the successful delivery of both DNA and peptide constructs, differences in transfection rates impacted the levels of immune response [79].

In an in vivo study, a DNA construct encoding the epitopes Nef60-84, Nef126-144, Vpr34-47, Vpr60-75, Gp16030-53, Gp160308-323, and P248-151 was utilized to produce the recombinant Nef-Vpr-Gp160-p24 polypeptide and Nef protein in *E. coli.* These proteins were then delivered into BALB/c mice using CPPs. The study assessed immune responses using various methods and found that the polyepitope DNA construct, which included conserved immunogenic epitopes from multiple HIV-1 subtypes, significantly enhanced both humoral and cellular immune responses. Notably, the study observed increased secretion of IgG2a, IgG2b, IFN-γ, and Granzyme B compared to other groups, indicating that the heterologous prime/boost regimens with both Nef-Vpr-Gp160-P24 and Nef antigens effectively stimulated immune responses. Additionally, CPPs demonstrated similar effectiveness to the Montanide adjuvant in stimulating immune responses. These results suggest that the recombinant polypeptide approach is more effective than synthetic peptides in generating robust immune responses [80].

To enhance the immunogenicity of antigens in both DNA- and protein-based HIV-1 vaccines, Rostami et al. [81] developed four CPPs for delivering genes and proteins, along with two adjuvants. The study aimed to boost the immune responses elicited by HIV-1 Nef, used as the antigen candidate. The CPPs included MPG (GALFLGFLGAAGSTMGAWSQPKKKRKV) and HR9 (CH5-R9-H5C) for gene delivery, and M918 (MVTVLFRRLRIRRACGPPRVRV) and Penetratin (P16: RQIKIWFQNRRMKWKK) for protein delivery. The adjuvants used were Hsp20 and Freund’s adjuvant [81].

The results indicated that MPG was more effective than HR9 for gene delivery in vitro. Moreover, the M918 peptide, especially when combined with MPG in a heterologous regimen, was more successful at stimulating cellular immune responses. This study demonstrated that the effectiveness of DNA or protein vaccines could be significantly enhanced through non-covalent interactions between CPPs (MPG, HR9, M918, and Penetratin) and either Nef or Hsp20-Nef DNA or protein, thereby boosting the overall immune response [81].

Overall, the development of an effective HIV-1 therapy faces numerous challenges, including resistance to current antiretroviral therapies, the persistence of latent viral reservoirs, and issues with drug toxicity and administration routes. Despite these obstacles, significant progress has been made, particularly with epitope-driven vaccine strategies targeting specific regions of the HIV-1 envelope glycoprotein. Recent studies have demonstrated the potential of combining peptide-based immunogens with advanced delivery systems, such as CPPs and recombinant protein approaches, to enhance immune responses. For instance, DNA constructs and peptide vaccines have shown varying degrees of success in generating robust immune responses, with CPPs playing a crucial role in efficient antigen delivery. Enhanced immunogenicity achieved through these strategies underscores the potential of combining multiple approaches to develop a more effective HIV-1 vaccine. Looking forward, continued research is essential to address the complex challenges of HIV-1 vaccine development. Future efforts should focus on optimizing antigen delivery methods, reducing the risk of resistance, and improving vaccine stability and efficacy.

### 3.3. Peptides Targeting RNA

**Figure 10:**
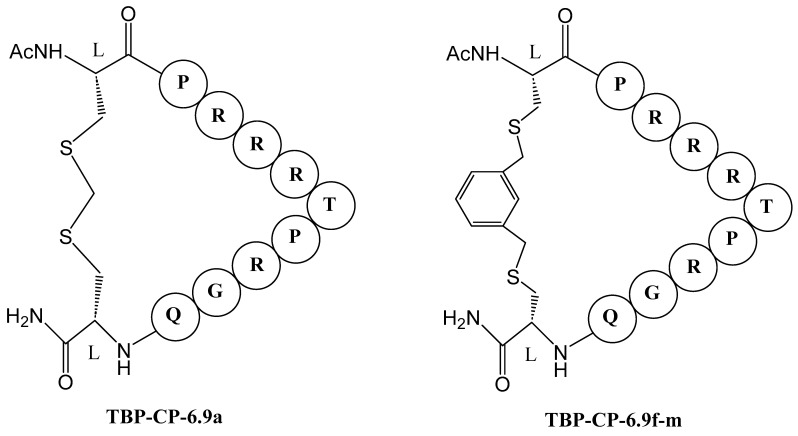
The chemical structures of TB-CP-6.9a and TBP-CP-6.9f-m.

Antiviral drug discovery often targets RNA molecules essential for viral replication. One effective approach involves using the cyclic peptide TB-CP-6.9a (Figure 10) to inhibit the interaction between the HIV-1 Tat protein and TAR (Trans-Activation Response) RNA. The research aimed to identify conserved RNA-binding interactions and evaluate how different cyclization linkers impact RNA binding and antiviral efficacy. In general, cyclic peptides constrained by methylene or naphthalene-based linkers exhibited significant antiviral activity by reducing HIV infectivity in cell culture. TB-CP-6.9a was developed from a TAR-binding loop derived from the TAR-binding protein (TBP) family. This peptide was designed based on the TBP scaffold. The TAR-binding cyclic peptide TB-CP-6.9a binds to HIV-1 TAR with a K_d_ of 3.6 ± 0.4 μM and effectively blocks the association between TAR and the Tat ARM peptide.

TB-CP-6.9f-m cyclic peptide (Figure 10) was shown to have a binding affinity to HIV-1 TAR RNA with 0.8 ± 0.1 μM. When the arginine was replaced with lysine, the K_d_ was increased to 8.2 ± 1.0 μM. Thus, TBP-derived cyclic peptides utilize an arginine-fork motif to specifically recognize the TAR major groove, distinguishing them from other TAR-targeting agents [82].

HIV-1 ribonucleic acid features a highly structured cis-acting element called the Rev Response Element (RRE), which spans 350 nucleotides and is crucial for viral replication. The natural Rev peptide binds to the RRE and assists in transporting it from the nucleus to the cytoplasm, where new virus particles are assembled. The synthetic peptide RSG1-2 (RRGSRPSGAERRRAAAA)–(DRRRGSRPSGAERRRRAAAA) mimics Rev’s function by binding to the RRE, thereby competing with Rev and blocking the transport of HIV-1 RNA. This interference ultimately inhibits viral replication [83].

Small interfering ribonucleic acids (siRNAs) are highly effective transcriptional inhibitors but require efficient delivery systems to penetrate target cells. The chimeric peptide FP-PTD combines an RNA-binding domain (PTD) with a cell-fusion peptide domain (FP) to enhance cellular uptake. FP-PTD is composed of the fusion peptide FP linked to PTD, KETWWETWWTEW-SQP-GRKKRRQRRR. This construct was effective in delivering specific siRNAs into HIV-1-susceptible and permissive cells. A combination of FP-PTD (15 mg) and siRNA (300 nM) was shown to be non-toxic to lymphocytic cells. The FP-PTD–siRNA complex effectively inhibited HIV-1 replication in target cells [84].

As described above, the exploration of peptide-based strategies targeting RNA has shown promising advancements in HIV-1 antiviral drug discovery. Looking ahead, the continued development of peptide-based RNA inhibitors should focus on optimizing their delivery, enhancing specificity, and minimizing potential off-target effects. Integrating these peptides with advanced delivery systems and exploring their efficacy in clinical settings will be crucial for advancing HIV-1 treatment options.

### 3.4. Peptide-Based Capsid Inhibitors

The constituents of the Gag precursor are HIV-1 capsid (CA) proteins. The Pr55Gag has a conserved structure but assembles into hexamers. This assembly forms a CA core with a conical shape that protects the virus’s RNA genome. Additionally, the Matrix (MA) proteins, which are distinct from Pr55Gag, are embedded in the viral membrane and play a crucial role in HIV-1 viral shell assembly. Both MA and CA could be promising targets for developing peptides that inhibit HIV-1 replication.

Mizuguchi et al. [85] evaluated the cell-penetrating CA peptides 1L, 2L, 6L, 8L, and 15L and demonstrated their anti-HIV activity against two HIV strains. Peptides 1L, 2L, 6L, and 8L exhibited strong anti-HIV activity against X4-HIV-1, while peptide 15L showed potent activity against R5-HIV-1, with 1L and 6L showing moderate activity. Peptides 1L, 2L, 6L, and 8L had EC_50_ values of 5.2, 7.8, 9.8, 15, and 11.2 µM, respectively, against HIV-1 (NL4-3 strain) in MT-4 cells. The strong anti-HIV activity of 15L is likely due to its sequence’s location in the H7 helix region and the linker connecting the *N*- and *C*-terminal domains of the CA proteins, highlighting the significance of this region in the CA protein’s conformational structure [85].

HIV-1 CA assembly occurs in tubes where CA monomers rearrange into a hexameric lattice, similar to the mature conical core. The distribution of HIV-1 mature-like particles was examined by Zhang et al. [86] using *i*, *i* + 7 hydrocarbon-stapled peptides. Three potent inhibitors of HIV-1 infection were studied: NYAD-36 (Ac-ISF-R8-ELLDYY-S5-ESGS-amide), NYAD-66 (Ac-ISF-R8-ELLDYY-S5-ED-amide), and NYAD-67 (Ac-ISF-R8-EWLQAY-S5-EdDE-amide), with IC_50_ values of 1.5 ± 0.17, 3.94 ± 0.32, and 3.88 ± 0.3, respectively. The peptides feature stapling sites marked by S5 [(S)-2-(4′-pentenyl)alanine] and R8 [(R)-2-(7′-octenyl)alanine]. In the presence of NYAD-36, NYAD-66, and NYAD-67 peptides, significant damage or disintegration of the formed CA tubes was observed [86].

The binding of the CA *C*-terminal domain (CTD) to human lysyl-tRNA synthetase (hLysRS) is crucial for the packaging of host cell tRNA (Lys,3), which acts as a primer for reverse transcription. Dewan et al. [87] screened cyclic peptides (CPs) for interaction with HIV-1 CA and identified two peptides: CP2 (cyclo(D-Ala-Trp-Gln-Fpa-Nle-D-Ala-Glu)-Lys) and CP4 (cyclo(D-Ala-Ile-Fpa-Arg-Tyr-Trp-D-Ala-D-Ala-Glu)-Lys). These peptides inhibited the in vitro interaction between hLysRS and CA. CP2 showed IC_50_ values of 0.16 ± 0.04 µM, while CP4 demonstrated IC_50_ values of 0.91 ± 0.1 µM for inhibiting LysRS/CA interactions, respectively. The low micromolar affinity of these CPs for HIV-1 CA-CTD highlights their potential as targets for anti-HIV peptide development [87].

Bocanegra et al. [88] reported that CAC1 (E^175^QASQEVKNWMTETLLVQNA^194^), its derivative CAC1M (SE^175^SAASSVKAWMTETLLVAN^193^TSS), and H8, which represents CA helix 8 (K^158^EPFRDYVDRFYKTLRAEQ^176^), effectively inhibited the assembly of the mature HIV-1 capsid in vitro. The H8 peptide, designed to mimic interfacial helices similar to CAC1, also showed the potential to inhibit HIV-1 capsid assembly and infection. CAC1 and CAC1M were used at concentrations of 170 µM, while the H8 peptide was used at 2.6 mM. CAC1 represents the sequence of helix 9 in CA and binds to the CTD, though it also tends to aggregate. The CAC1-derived peptide CAC1M includes several amino acid substitutions and short *N*- and *C*-terminal extensions, which are believed to reduce aggregation, increase solubility, and enhance helical structure. Additionally, these modifications appear to limit the dimerization affinity within the CTD domain [88].

Infectious HIV-1 is generated through the assembly of Gag polyproteins into immature particles, which then mature into capsids following the proteolytic disassembly of the Gag shell. Sticht et al. [89] identified a 12-mer peptide (ITFEDLLDYYGP) as a capsid assembly inhibitor that binds to the CA domain of Gag, leading to inhibition of both immature and mature-like capsid particle assembly in vitro [89].

Overall, the development of peptide-based capsid inhibitors represents a promising avenue for HIV-1 therapy by targeting the virus’s core assembly and maturation processes. Research has demonstrated that peptides targeting the HIV-1 CA protein, such as the CPPs and hydrocarbon-stapled peptides, can effectively inhibit viral replication by disrupting capsid assembly and stability. Additionally, peptides that interfere with crucial interactions, such as those between the CA *C*-terminal domain and human lysyl-tRNA synthetase, further underscore the potential of peptide-based inhibitors. Exploring combination therapies that leverage peptide-based capsid inhibitors with other antiviral strategies could also offer synergistic effects. As our understanding of HIV-1 capsid dynamics and peptide interactions deepens, these innovative approaches hold the potential to significantly advance the development of effective antiviral treatments.

### 3.5. Defensins

Peptides act as antiviral host defense agents through various mechanisms. One notable example is defensins, which are 30–40 amino acid peptides characterized by a β-sheet structure stabilized by three disulfide bonds, along with cationic and amphipathic properties. Human α-defensins 1–3 and human β-defensins 2 and 3 are known to inhibit the replication of CCR5- and CXCR4-tropic HIV-1 strains [90,91,92,93,94,95].

One member of the cathelicidin protein family is hCAP-18, which can be processed into 16 different fragments. Among these, LL-37 is a 37-amino acid cationic peptide produced through proteinase 3-mediated cleavage at the *C*-terminal end of hCAP-18. LL-37’s amphipathic nature allows it to integrate into lipid bilayers, thereby protecting it from proteolytic degradation [77,96].

Both human cathelicidin LL-37 and its fragments LL13-37 and LL17-32 have been shown to inhibit HIV-1 reverse transcriptase with IC_50_ values of 15 μM, 7 μM, and 70 μM, respectively. LL13-37 exhibits greater potency in inhibiting HIV-1 reverse transcriptase and protease compared to LL-37. Conversely, LL13-32, which consists of 26 amino acids, demonstrates weaker inhibition of HIV-1 reverse transcriptase compared to LL-37 but shows stronger inhibition of HIV-1 protease. This suggests that peptide fragments 14–16 and 33–36 contribute to LL-37’s inhibitory effect on HIV-1 reverse transcriptase, while these fragments do not significantly affect HIV-1 protease inhibition. Additionally, LL-37 has been found to inhibit HIV-1 protease [90,97,98,99,100].

Defensins and related peptides exemplify the diverse mechanisms through which host-derived peptides can act as antiviral agents. The study of defensins and cathelicidins offers valuable insights into the development of novel antiviral therapies. Future research should focus on exploring the structural and functional relationships between peptide sequences and their antiviral activities. Understanding how specific peptide fragments contribute to antiviral efficacy could lead to the design of more potent and selective inhibitors. Additionally, investigating the potential synergistic effects of defensins with other antiviral agents may offer new avenues for therapeutic development.

### 3.6. Plant-Derived Anti-HIV Peptides

The use of peptides in antiviral research is well-documented across various resources. Given that medicinal plants are a rich source of bioactive metabolites, screening plant extracts for natural peptides with anti-HIV activity is a key strategy for developing effective peptides against HIV.

Cyclotides are plant-derived peptides consisting of approximately 30 amino acid residues, featuring a cyclic backbone and a cystine knot [101], which provides resistance to thermal, chemical, and enzymatic degradation. These peptides have shown promising potential in drug development as molecular scaffolds due to their ability to disrupt protein–protein interactions (PPIs), cross cellular membranes, and retain biological activity. Additionally, engineered cyclotides like those derived from MCoTI-I/II have been developed as CXCR4 antagonists, p53 activators, and imaging agents [102].

The structural features of cyclotides contribute to their anti-HIV-1 activity due to their folding and cyclization at the *N*- and *C*-termini, forming a peptide bond and regional hydrophobicity. Cyclotides are particularly effective against the viral envelope, which is a key target due to its hydrophobic properties [103]. It was discovered that the integrity of the cyclotide backbone is linked to their higher hydrophobicity and anti-HIV activity. The anti-HIV effects of cyclotides from the bracelet and Möbius subfamilies varied based on the positioning of hydrophobic and charged residue patches on their surfaces [104].

Cyclotides have been extracted from plants in the Violaceae, Cucurbitaceae, and Rubiaceae families, where they serve as defense agents [105]. Screening subfractions of cyclotides extracted from *Viola tricolor* demonstrated an inhibitory effect on HIV-1 infection, with IC_50_ values ranging from 0.6 to 11.2 µg/mL [106]. Transcriptomic and proteomic analyses of *Viola tricolor* revealed the presence of 168 cyclotides, suggesting that the Violaceae family may contain around 150,000 different cyclotides [107,108,109].

Aboye et al. (2012) [110] reported the design and synthesis of a novel cyclotide-based compound targeting the CXCR4 receptor to inhibit HIV-1 entry. By grafting modified versions of the CXCR4-binding peptide CVX15 into the loop of the cyclotide MCoTI-I, the researchers developed MCo-CVX-5c, a potent CXCR4 antagonist and HIV-1 entry blocker with an EC_50_ of approximately 2.0 nM. This engineered cyclotide exhibited high specificity, stability in human serum (half-life of 62 h), and low cytotoxicity [110].

Lesniak et al. (2017) [111] evaluated the in vivo potential of the engineered cyclotide MCo-CVX-5c as a CXCR4 antagonist for molecular imaging applications. A [^64^Cu]-DOTA-labeled version of the cyclotide demonstrated high uptake and retention in CXCR4-expressing glioblastoma tumors in mice, as observed through PET-CT imaging. The radiolabeled cyclotide showed targeting specificity, which was confirmed by blocking experiments using non-radioactive MCo-CVX-5c and the FDA-approved CXCR4 inhibitor AMD3100 [111]. The impact of lipidation on the pharmacokinetic profile and biological activity of MCo-CVX-5c was studied by Chaudhuri et al. (2023) [112]. Although MCo-CVX-5c is rapidly cleared through renal elimination, lipidation with palmitic acid (MCo-CVX-5c-PP) significantly extended its half-life while maintaining or slightly enhancing its bioactivity, including CXCR4 inhibition, HIV-1 infectivity reduction, and lymphoma cell cytotoxicity. However, lipidation with octadecanedioic acid (MCo-CVX-5c-OP) yielded a much longer half-life but drastically reduced bioactivity.

Crude extracts of *Chassalia pravifolia* exhibited anti-HIV activity. Bioassay-guided isolation identified two macrocyclic peptides: Circulin A (VCYRNGEPCGESDVWIPCISAALGCSCK + NK) and Circulin B (NGVIPCGESCVFIPCISTLLGCSCKNKVCYR) B [113].

HIV-1 reverse transcriptase (HIV-1 RT), which catalyzes the conversion of viral RNA genomes into DNA, is a key target for anti-HIV therapies. Seetaha et al. [114] investigated hydrolyzed peptides extracted from Asian medicinal plants, synthesizing two peptides from the fruit peel of *Quercus infectoria*: AIHIILI and LIAVSTNIIFIVV. These peptides were assessed for their ability to inhibit HIV-1 RT, with IC_50_ values of 274 ± 5.10 nM and 236.4 ± 7.07 nM, respectively [114].

The future of antiviral peptide research should continue to harness the rich diversity of plant-derived peptides. Comprehensive screening of medicinal plants for peptides with anti-HIV activity is crucial for identifying new therapeutic candidates. Research should focus on optimizing the extraction and synthesis of these peptides to enhance their efficacy and stability. Further studies are also needed to elucidate the mechanisms by which these peptides interact with viral components and to assess their safety and effectiveness in clinical settings.

## 4. Conclusions

HIV-1 remains a significant global health challenge despite advancements in treatment. The virus’s complexity and the persistence of latent infections highlight the limitations of current therapies. While antiretroviral therapy (ART) has transformed HIV from a fatal condition to a manageable chronic disease, achieving complete eradication remains elusive.

Peptide-based therapies, including ENF and ABT, represent promising advancements in HIV treatment. These drugs, which target crucial stages of viral entry and fusion, have shown efficacy in managing resistant strains of HIV-1 and have been instrumental in improving patient outcomes. ENF’s approval marked a significant milestone, and its subsequent modifications, such as glycosylation and PEGylation, have enhanced its pharmacokinetic properties. Similarly, ABT, with its longer half-life and broad-spectrum activity, addresses some of the limitations associated with earlier treatments.

Emerging peptide-based inhibitors, including those targeting the *N*-heptad repeat (NHR) and *C*-heptad repeat (CHR) regions of gp41, have demonstrated high potency and specificity. The development of novel peptides such as lipopeptide-19 (LP-19) showcases the potential for these agents to overcome resistance and improve treatment efficacy. The continued exploration of peptide sequences and their conjugates with lipids or other modifications has furthered our understanding of their antiviral mechanisms and therapeutic potential.

The research presented highlights significant advancements in the development and application of peptide-based strategies for HIV-1 inhibition and vaccine development. The diverse approaches, ranging from peptide fusion inhibitors, cyclic peptides, and α/β-peptides to plant-derived and synthetic peptides, underscore the potential of these molecules in targeting various stages of the HIV-1 life cycle. Key findings include the efficacy of PEGylated C34 peptides, which demonstrated broad-spectrum anti-HIV-1 activity with enhanced stability due to PEGylation. The study also highlighted the potency of peptides targeting the CHR region of gp41, as well as the significant impact of peptide crosslinker positioning on binding affinity and inhibition effectiveness.

The effectiveness of trimeric synthetic peptides, particularly the N36 peptide, in enhancing stability and antiviral activity underscores the potential for developing peptide-based inhibitors with improved therapeutic profiles. The observed synergistic effect of combining different fusion inhibitors, such as Hcs6ERE and HP23, indicates the benefit of multi-target approaches in overcoming viral resistance and enhancing antiviral efficacy.

Additionally, the development of peptides targeting the PBD and co-receptor interactions represents a promising strategy for HIV-1 inhibition. The findings on the cyclic and all-D amino acid analogs of the V3 loop peptide further emphasize the complexity of co-receptor recognition and the potential for designing versatile inhibitors that accommodate viral mutations.

## 5. Future Perspectives

When investigating new peptides as potential anti-HIV agents, it is essential to weigh their advantages and disadvantages carefully. Key factors for selecting an effective anti-HIV peptide include high activity, low toxicity, and an appropriate administration route. Furthermore, the peptide’s specificity, stability, selectivity, and small size are crucial attributes that influence its effectiveness as an anti-HIV drug. Furthermore, peptides offer several advantages over antibodies, particularly in resource-limited settings where maintaining a cold temperature for biologics poses significant challenges. Peptides can be rapidly synthesized through well-established chemical methods, reducing production costs and enabling faster scalability. Unlike antibodies, which often require cold storage to maintain stability, many peptides are stable at room temperature, simplifying their storage and transport without the need for refrigeration. This makes peptide-based therapies more accessible in disadvantaged regions with limited healthcare infrastructure. The ongoing research and development in peptide-based HIV-1 therapies hold promise for several key areas of advancement:

**Optimization of Peptide Stability and Delivery**. Peptides often face challenges related to stability, bioavailability, and effective delivery. Future research should focus on enhancing the stability and bioavailability of peptide-based drugs. While PEGylation has shown promise in extending peptide stability and efficacy, further optimization is needed to balance the size and pharmacokinetics of PEGylated peptides. Future research should explore different PEG sizes and configurations to maximize therapeutic benefits. Advances in drug delivery systems, such as nanoparticle conjugation [115] and improved CPP formulations will be crucial for effective therapeutic application. Enhancing peptide stability through structural modifications, such as cyclization or stapling, alongside developing effective delivery systems, will be crucial for advancing peptide-based therapies. Innovations in peptide formulation and delivery could improve bioavailability and therapeutic outcomes.

**Broad-Spectrum and Multivalent Peptides**. The success of trimeric N36 peptides in enhancing stability and activity suggests that further exploration of trimeric designs for other peptide inhibitors could yield significant advancements. Investigating various trimerization strategies and carrier molecules could lead to more effective peptide-based therapies. Developing peptides with broad-spectrum activity against various HIV-1 strains and subtypes will be essential. Combining multiple inhibitory mechanisms within a single peptide or construct could improve the efficacy of treatments. Such multi-target strategies may help reduce the likelihood of resistance development and enhance therapeutic efficacy across diverse HIV-1 strains.

**Personalized Peptide Therapies**. Advances in genomics and proteomics could lead to more personalized approaches to HIV treatment. Personalized medicine has begun to influence various therapeutic areas, but peptide-based HIV therapies are still evolving in this context. Tailoring peptide-based therapies to individual patient profiles, including HIV-1 strain and resistance patterns, may enhance therapeutic outcomes. Personalized medicine approaches could help address the variability in patient responses to HIV treatments.

**Combination Therapies.** The synergistic effects observed with peptide combinations, such as Hcs6ERE and HP23, highlight the potential of using multi-target approaches to enhance antiviral efficacy. Future studies should focus on optimizing combination therapies to target multiple stages of HIV-1 entry and fusion, potentially reducing the likelihood of resistance development. Combining peptide-based inhibitors with existing ART regimens could provide synergistic effects, potentially improving treatment outcomes. 

**Targeting HIV Reservoirs.** Addressing latent HIV reservoirs remains a critical challenge. Future research should explore how peptide-based inhibitors can be integrated into strategies to eradicate these reservoirs. Novel peptides that specifically target latent HIV-1 or enhance the reactivation of dormant virus could be key to achieving a functional cure.

**Coreceptor-Specific Inhibitors.** Peptides that selectively interact with co-receptors CXCR4 and CCR5 have shown promise. Given the selective interaction of certain peptides with CXCR4 or CCR5, further development of co-receptor-specific inhibitors could improve treatment options for patients with different HIV-1 strains. Research should focus on designing peptides that can effectively target both co-receptors and overcome co-receptor mutations.

**Exploration of New Targets and Mechanisms**: Continued exploration of novel peptide targets, such as additional viral proteins or cellular co-factors, will be vital for identifying new avenues for HIV-1 inhibition. This strategy can be accomplished using bioinformatic and in silico studies. The integration of in silico studies and computational drug discovery plays a crucial role in advancing peptide-based anti-HIV therapy. Traditional methods for screening active anti-HIV peptides, relying on conventional separation and purification techniques, are often slow and lack precision in identifying specific modes of action. The challenges associated with extracting bioactive natural and microbial compounds have further driven the adoption of bioinformatics tools. Techniques such as proteomics, genomics, and peptidomics contribute significantly to the pharmacokinetic and pharmacodynamic analysis of active peptides. The process typically begins with computer-aided sequence exploration, followed by active site prediction, molecular docking, and molecular dynamics simulations, which can work together to facilitate a faster and more accurate investigation of potential anti-HIV peptides [116].

**Human Organoid for Evaluation of Peptides.** The potential of human organoids as alternatives to animal models for testing peptides in anti-HIV research offers a platform for personalized medicine by integrating individual genotypes. One key driver for exploring HIV cure strategies is the persistence of viral reservoirs in specific tissue sites, such as the central nervous system (CNS). Brain organoids, derived from human induced pluripotent stem cells (hiPSCs) and developed either spontaneously or with growth factors during immature or mature stages, have emerged as advanced models for studying neurovirulent infections, including HIV. These organoids replicate the 3D structure and cellular interactions of the human brain, making them ideal for investigating how viral reservoirs persist and resist clearance. Furthermore, they enable research into improving viral penetration and immune surveillance within the CNS. Personalized approaches to treatment can also be tested through these models, advancing the development of curative strategies that target viral reservoirs while minimizing neuroinflammation. Brain organoids thus represent an ethical, physiologically relevant tool to explore both disease mechanisms and individualized therapies [117,118].

**Clinical Trials and Efficacy Studies**: Rigorous clinical trials are necessary to evaluate the safety and efficacy of peptide-based therapies in human populations. These studies will provide critical data on dosing, potential side effects, and therapeutic benefits. As peptide-based inhibitors advance toward clinical applications, ongoing monitoring of viral resistance and efficacy in diverse patient populations will be essential. Clinical trials should aim to evaluate these inhibitors’ long-term effectiveness and safety, ensuring they can be integrated into current treatment regimens. Efforts to reduce the global burden of HIV must continue, with a focus on addressing disparities in access to treatment and prevention. Innovations in peptide-based therapies should be coupled with global health strategies to improve access to care, especially in high-prevalence regions such as sub-Saharan Africa.

**Vaccine Development and Peptide-Based Immunogens**: The development of peptide-based vaccines with enhanced immunogenicity and advanced delivery methods holds potential for effective prophylactic and therapeutic vaccines against HIV-1.

Overall, the advancements in peptide-based HIV-1 inhibitors outlined in this study provide a promising foundation for future research and development. By addressing the challenges and leveraging the opportunities identified, researchers can contribute to developing more effective and durable therapies for HIV-1. While significant strides have been made in HIV treatment through peptide-based inhibitors and other antiretroviral therapies, ongoing research and innovation are essential. Addressing the limitations of current therapies, exploring new peptide strategies, and focusing on global health implications will be crucial for advancing HIV treatment and ultimately achieving a cure.

## Figures and Tables

**Table 1:** Epidemiology of HIV infection in 2022: UNAIDS data 2023.

Country	New Infections (per 1000 Uninfected Population)	AIDS-Related Deaths	People Living with HIV
Global	0.17 (0.13–0.23)	630,000 (480,000–880,000)	39,000,000 (33,100,000–45,700,000)
African Region	0.57 (0.41–0.8)	380,000 (300,000–540,000)	25,600,000 (21,600,000–30,000,000)
Eastern and Southern Africa	1.07 (0.78–1.45)	240,000 (190,000–360,000)	20,400,000 (17,200,000–24,100,000)
Western and Central Africa	0.26 (0.17–0.39)	140,000 (110,000–180,000)	5,100,000 (4,500,000–5,900,000)
Region of the Americas	0.16 (0.13–0.19)	41,000 (31,000–54,000)	3,800,000 (3,400,000–4,300,000)
Southeast Asia Region	0.06 (0.04–0.08)	85,000 (62,000–120,000)	3,900,000 (3,400,000–4,600,000)
European Region	0.2 (0.16–0.23)	52,000 (40,000–65,000)	3,000,000 (2,600,000–3,300,000)
Eastern Mediterranean region	0.07 (0.06–0.1)	20,000 (16,000–27,000)	490,000 (420,000–60,0000)
Western Pacific Region	0.07 (0.05–0.1)	51,000 (30,000–80,000)	2,200,000 (1,700,000–2,800,000)

Source: UNAIDS/WHO estimates, 2023.

## Data Availability

Not applicable.
